# Isolated Ascending Colon Gangrene With Persistent Ascending Mesocolon: A Case Report

**DOI:** 10.7759/cureus.29978

**Published:** 2022-10-06

**Authors:** Mahalakshmi Ashokkumar, Barath Prashanth Sivasubramanian, Sandhya R Palit, Vikramaditya Samala Venkata, Uma D Dhanasekaran, Mohamed Afzal

**Affiliations:** 1 General Surgery, KAP Viswanatham Government Medical College, Trichy, IND; 2 Internal Medicine, Employees' State Insurance Corporation Medical College and Postgraduate Institute of Medical Sciences and Research, Chennai, IND; 3 General and Colorectal Surgery, Employees' State Insurance Corporation Medical College and Postgraduate Institute of Medical Sciences and Research, Chennai, IND; 4 Internal Medicine, Cheshire Medical Center, Dartmouth-Hitchcock, Keene, USA

**Keywords:** emergency laparotomy, colon gangrene, persistent mesocolon, adult malrotation, volvulus

## Abstract

Persistent mesocolon is a rare embryological anomaly that occurs when the primitive dorsal mesocolon fails to fuse with the posterior lateral parietal peritoneum allowing for volvulus of the cecum and colon. In malrotation of the gut, the normal rotation of the embryonic gut is arrested or disturbed during in-utero development.

To our knowledge, this is the first case of isolated colon gangrene with persistent ascending mesocolon to be reported, although earlier studies have documented cases of persistent mesocolon of both ascending and descending mesocolon. This case had signs of acute intestinal obstruction and peritonitis, and preoperative imaging was suggestive of sigmoid volvulus. Explorative laparotomy revealed a hugely dilated and gangrenous ascending colon; the fixed part of the small intestine was found in the subhepatic space, and the hepatic flexure was at a level below the transpyloric plane in the midclavicular line. Findings were suggestive of isolated colon gangrene with persistent ascending mesocolon and malrotation of the gut. Right hemicolectomy with terminal ileostomy was performed and postoperative follow-up showed no complications.

For a young patient with no prior co-morbidities, a volvulus of ascending mesocolon diagnosis was deemed unlikely because ascending colon is a retroperitoneal organ. The medial position of ascending mesocolon and small bowel loops to the right side was a crucial intra-operative clue for diagnosis. Such cases are difficult to diagnose pre-operatively through imaging alone, hence explorative laparotomy becomes necessary. Intra-operative findings led to performing a right hemicolectomy and ileostomy to relieve the obstruction. Therefore, among other congenital reasons for intestinal obstruction, surgeons should consider persistent mesocolon and volvulus as differential diagnoses when evaluating young patients. Emergent surgery is the only approach to address this.

## Introduction

The frequency of occurrence of persistent ascending mesocolon is about 2.4% [1]. In malrotation of the gut, the normal rotation of the embryonic gut is arrested or disturbed during in-utero development. The prevalence of malrotation in adults is unknown, but colorectal screening by computed tomography (CT) colonography has suggested a prevalence of 0.17% [2]. Because of its rarity, the diagnosis of intestinal malrotation in adult patients is often delayed and therefore associated with significant morbidity [3,4].

Persistent ascending mesocolon is a rare embryological anomaly that occurs in the final process of intestinal development during organogenesis. The primitive dorsal mesocolon fails to fuse with the parietal peritoneum in the fifth month of gestation. Volvulus and gangrene can arise from persistent mesocolon because it allows for abnormal colon motility [5]. Only scant evidence of chronic mesocolon exists, but it has been recognized alongside colonic varices and intussusception [6,7]. Kanai et al. reported the first case of colonic varices complicated by persistent mesocolon of ascending and descending mesocolon [6]. But in this instance, mesocolon has never been discovered in a patient with colon gangrene. Cancer and volvulus are the two most frequent causes of large bowel obstruction [8-14]. The majority of benign large bowel obstructions (15-20%) are caused by volvulus [8]. Here, we present a case of colon gangrene that is highly unusual, together with persistent ascending mesocolon and gut malrotation, that was found in a young patient with no history of concomitant conditions. Volvulus is a common cause of bowel obstruction; however, since the ascending colon is a retroperitoneal organ, it is uncommon to diagnose volvulus of the ascending mesocolon. Furthermore, this is understandable given that persisting mesocolon permits abnormal colon motility [5]. 

## Case presentation

**Figure 1:**
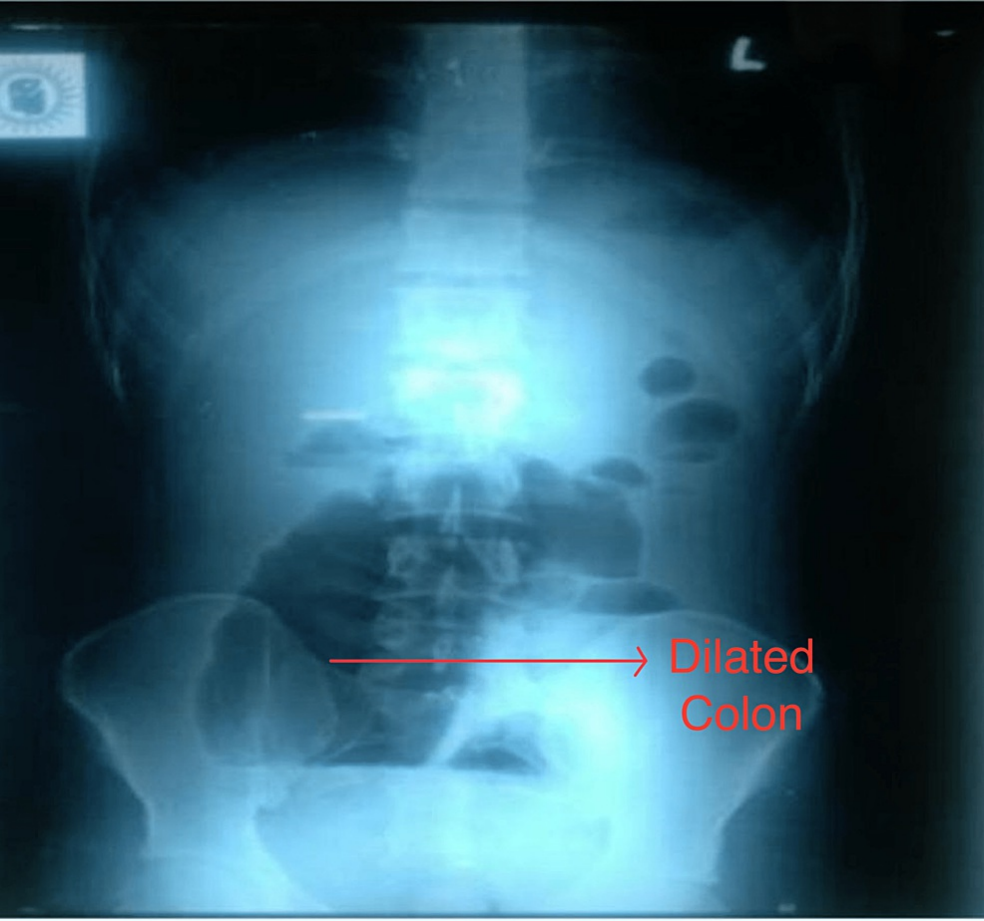
Dilated large bowel loops on X-ray of the erect abdomen

A 20-year-old male was admitted for abdominal pain for three days prior to presentation, accompanied by several episodes of vomiting, and had not passed motion and flatus for three days with no prior co-morbidities. On physical examination, the patient was afebrile, with no pallor, and mild dehydration was present. Vital signs showed normal temperature, heart rate of 120 beats/minute, normal respiratory rate, and blood pressure. On physical exam, the abdomen was distended with diffuse tenderness, rigidity, guarding, and absent bowel sounds. Per rectal examination was normal. Complete blood count and other routine investigations were found to be within normal limits. The patient was commenced on intravenous fluids and a chest X-ray and X-ray of the erect abdomen showed dilated large bowel shadow on the right side of the abdomen with multiple air-fluid levels indicating acute intestinal obstruction (Figure 1).

**Figure 2:**
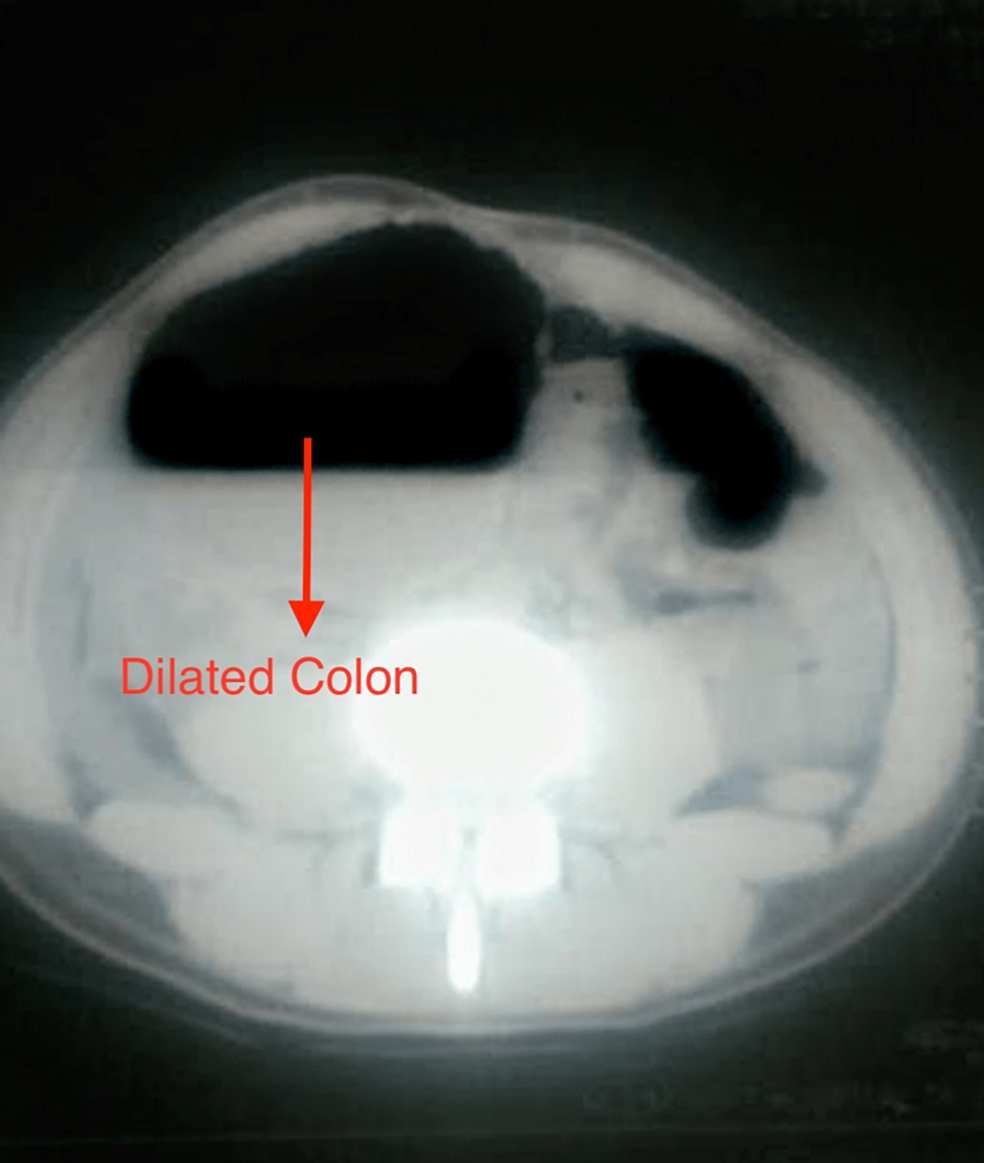
CT finding showing dilated large bowel on the right side

The ultrasound of the abdomen showed dilatation of the large bowel loop on the right side of the lower abdomen and in the loops of the small intestine, suggestive of sigmoid volvulus. The CT scan of the abdomen without contrast revealed sigmoid volvulus (Figure 2).

**Figure 3:**
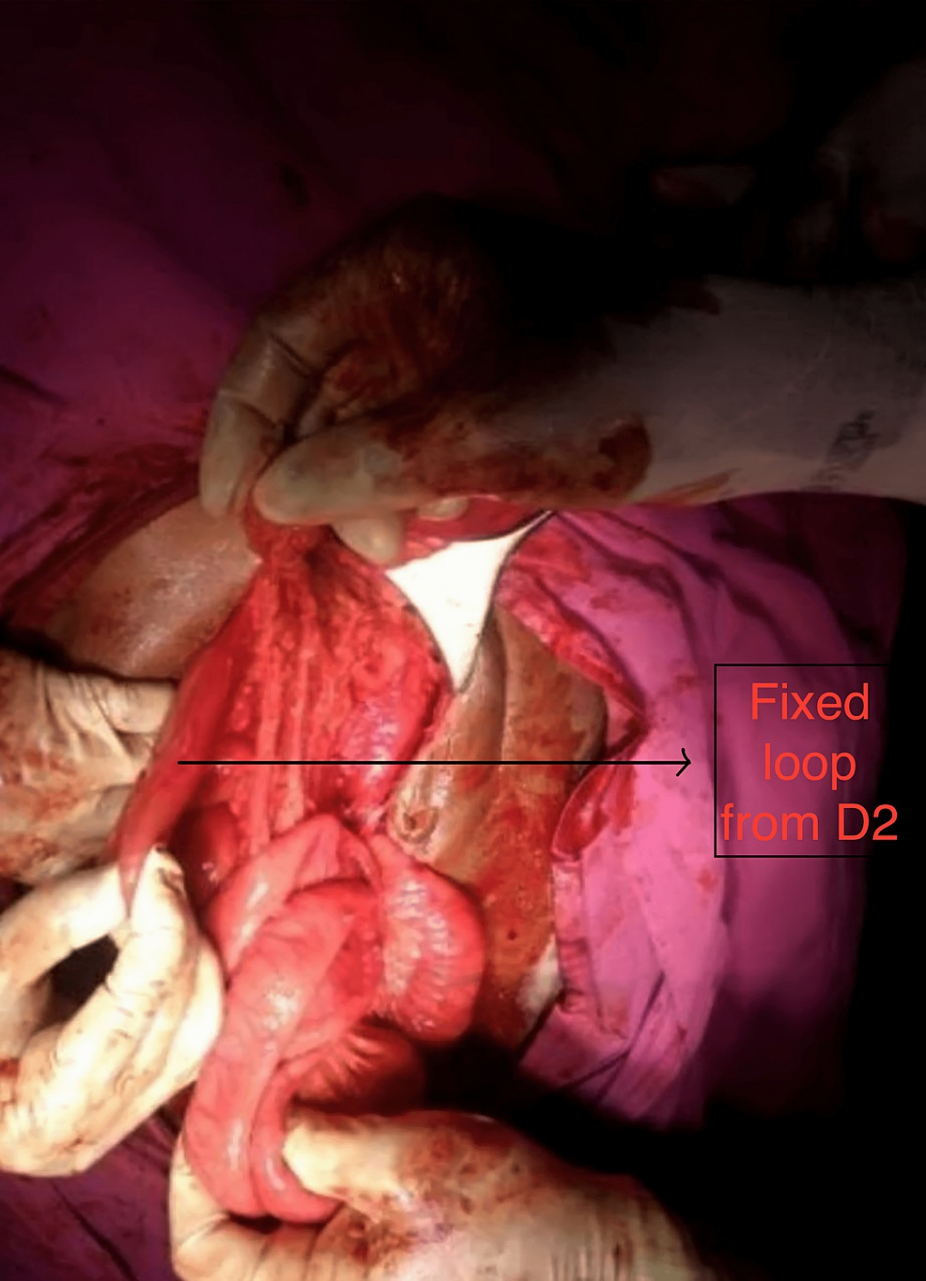
Fixed loop arising from D2 The site of the fixture is indicated by the arrow

Differential diagnoses of volvulus of the colon, intussusception of the colon, and mesenteric ischemia were made. The patient was taken for emergency laparotomy due to acute intestinal obstruction with features of peritonitis. A midline incision was made and the ascending colon was found to be freely mobile, hugely dilated, and gangrenous. The ileum, ileocecal junction, and appendix were normal. The cecum was found to be dilated and the part below the ileocecal junction was normal. Hepatic flexure was at the level below the trans-pyloric plane along the midclavicular line. Duodenojejunal flexure was not found at the usual place, instead fixed part was seen on the right side. Small bowel loops were occupying the subhepatic space (Figure 3).

A right hemicolectomy with terminal ileostomy was done. The post-operative period was uneventful. The patient was started on oral fluids on the second postoperative day and sutures were removed on the 10th postoperative day. The patient was readmitted after six weeks. Ostomy reversal with ileo-transverse anastomosis was performed.

## Discussion

This is a rare case of isolated ascending colon gangrene with persistent ascending mesocolon, which initially presented as acute intestinal obstruction and features suggestive of colon gangrene due to persistent mesocolon and malrotation of the gut. A diagnosis of volvulus of ascending colon is not common, as the mesocolon is a retroperitoneal organ.

The ascending colon is usually surrounded by peritoneum on three sides and is a retroperitoneal structure without a mesocolon. However, if the primitive dorsal mesocolon fails to fuse with the parietal peritoneum in the fourth through the fifth month of gestation, it leads to the formation of persistent ascending mesocolon resulting in the mobility of the ascending colon and increasing changes of volvulus [5]. Volvulus commonly occurs in those portions of the colon possessing a mesentery including the sigmoid, cecum, and transverse colon. There is an increased risk of torsion due to this increased mobility of ascending colon [5]. Kanai et al. reported the first case of colonic varices complicated by persistent mesocolon of ascending and descending mesocolon. The persistent mesocolon may have induced local hypertension of the intestinal venous system, leading to colonic varices [6]. Ongom et al. reported a case of anal protrusion of an ileocolic intussusception with persistent ascending and descending mesocolon. The intussusceptum was incompletely reduced and a right hemicolectomy with ileo-transverse colonic anastomosis was performed. Histopathological examination revealed no preexisting pathologic lesion as a lead point [7]. Tsuruta et al. published a paper on 13 cases of persistent descending colon [1]. This is the first case of isolated colon gangrene with persistent ascending mesocolon to be reported. For a young patient with no prior comorbidities, a diagnosis of volvulus of ascending mesocolon is unlikely because ascending colon is a retroperitoneal organ. The medial position of ascending mesocolon and small bowel loops to the right side might serve as a diagnostic clue for the presence of persistent mesocolon. Due to technical difficulties at our institution, we were only able to get a CT abdomen without contrast. The markedly dilated bowel loop depicted on radiography was suggestive of volvulus, but the exact obstruction level was hard to be accurately identified. Contrast-enhanced CT abdomen is the diagnostic test of choice. A contrast-enhanced CT can show the presence, level, and degree of obstruction as well as signs of strangulation [15].

## Conclusions

To our knowledge, this is the first reported case of persistent ascending mesocolon leading to colon gangrene. Surgeons should consider the possibility of developmental anomalies like persistent ascending mesocolon and volvulus in a young patient with colon gangrene. 
